# Parameters of rotational thromboelastometry in patients with moderate and severe psoriasis: a pilot study

**DOI:** 10.1007/s11239-025-03140-z

**Published:** 2025-07-01

**Authors:** Martin Jozef Péč, Tomáš Bolek, Jakub Benko, Jakub Jurica, Monika Péčová, Miroslava Drotárová, Jana Žolková, Karolína Vorčáková, Ingrid Škorňová, Marián Grendár, Juraj Sokol, Tatiana Hurtová, Matej Samoš, Marián Mokáň

**Affiliations:** 1 Department of Internal Medicine I, Jessenius Faculty of Medicine in Martin, Comenius University in Bratislava, Martin, 036 59 Slovakia; 2 Department of Hematology and Transfusiology, Jessenius Faculty of Medicine in Martin, Comenius University in Bratislava, Martin, 036 59 Slovakia; 3 Oncology Centre, Teaching Hospital Martin, Martin, 036 59 Slovakia; 4 Department of Dermatovenerology, Jessenius Faculty of Medicine in Martin, Comenius University in Bratislava, Martin, 036 59 Slovakia; 5 Department of Bioinformatics, Biomedical Centre Martin, Jessenius Faculty of Medicine in Martin, Comenius University in Bratislava, Martin, 036 01 Slovakia; 6 Department of Infectology and Travel Medicine, Jessenius Faculty of Medicine in Martin, Comenius University in Bratislava, Martin, 036 59 Slovakia; 7 Division of Acute and Interventional Cardiology, Department of Cardiology and Angiology II, Mid-Slovakian Institute of Heart and Vessel Diseases (SÚSCCH, a.s.) in Banská Bystrica, Banská Bystrica, 974 01 Slovakia

**Keywords:** Hypercoagulation, Psoriasis, Venous thromboembolism, Rotational thromboelastometry

## Abstract

**Introduction:**

Several studies have repeatedly described an increased risk of thrombotic complications in patients with severe psoriasis. Rotational thromboelastometry (ROTEM) is a viscoelastic hemostatic test that allows sophisticated in-vivo evaluation of hemostasis in whole blood samples. This study aimed to assess hemostatic changes in psoriatic patients using ROTEM.

**Methods:**

This pilot, observational, prospective study included 62 patients with moderate and severe psoriasis and a control group of 61 healthy blood donors. Blood samples were tested using the ROTEM Gamma analyser (Pentapharm GmbH, Munich, Germany) with INTEM, EXTEM, and FIBTEM reagents. We measured clotting time (CT), clot formation time (CFT), maximum clot firmness (MCF), amplitude at 10 and 20 min post-CT (A10, A20) and alpha angle.

**Results:**

The psoriatic patient group consisted of 31 men and 31 women, while the control group included 34 men and 27 women. Comparing patients with psoriasis and the control group, we identified statistically significant differences in the parameters: CT-EXTEM (74.8±1.4s vs. 69.7±1.4s; *p* < 0.05), MCF-EXTEM, A10-EXTEM, A20-EXTEM, CFT-INTEM, A10-INTEM, A20-INTEM, alfa-INTEM, CT-FIBTEM (67.3±1.2s vs. 62.8±1.2s; *p* < 0.05), CFT-FIBTEM and alfa-FIBTEM.

**Conclusions:**

In this pilot study, compared to the controls, patients with moderate to severe psoriasis exhibited a shift in hemostasis towards a procoagulant state in ROTEM analysis.

## Graphical Abstract

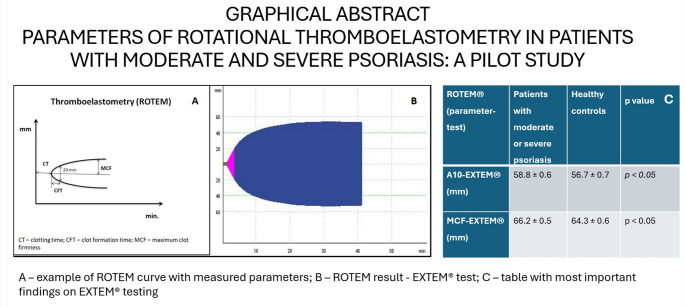

## Introduction

Psoriasis is a chronic papulosquamous immune-mediated inflammatory skin disorder with a prevalence ranging from 0.51 to 11.43% ^1^. Psoriasis occurs at any age and results in a significant burden on individuals and society. Immunological and genetic studies have identified interleukins 17 and 23 as key factors in the pathogenesis of psoriasis. Currently, psoriasis cannot be cured, but management should focus on minimizing physical and psychological harm to patients in the early stages of the disease, identifying and preventing associated multimorbidity using a personalized treatment approach [2]. Psoriasis is associated with a decrease in the quality of life and various comorbidities, including depression, psoriatic arthritis, cardiometabolic syndrome and an increased risk of thromboembolic events [3–5]. The underlying pathophysiological mechanisms linking psoriasis to these comorbidities are still unclear. Hemostasis involves a complex interaction between the vascular endothelium, platelets, and the coagulation cascade. In psoriasis, various studies reported an increased risk of venous thromboembolism (VTE) [6–8]. Independent predictors for VTE in patients with psoriasis included older age, diabetes mellitus and corticosteroid therapy [9]. It is believed that platelets and chronic subclinical inflammation have a major impact on hemostatic disturbances in patients with psoriasis [10, 11]. Traditional coagulation tests, however, may not fully capture these nuanced alterations in hemostasis. This has led to an increased interest in the use of advanced hemostatic assessment tools such as rotational thromboelastometry (ROTEM). ROTEM is a viscoelastic method measuring the mechanical properties of a coagulum (viscoelastic properties) during clot formation, stabilization and lysis in real-time [12]. Along with thromboelastography (TEG), ROTEM is a coagulation method that, unlike standard coagulation tests such as prothrombin time and activated partial thromboplastin time, uses a whole blood sample, not only blood plasma, for examination [13]. ROTEM offers detailed insights into the functional status of the coagulation system, making it a valuable tool for assessing hemostatic disorders in various clinical contexts and reflecting in vivo hemostasis. According to published research, ROTEM has been applied to heart surgery, liver transplants, ischemic heart disease, trauma, bleeding disorders, COVID-19, management of high-risk pregnant patients and testing for direct oral anticoagulants [14–19]. In dermatology and rheumatic settings, ROTEM is not utilized yet. Our study’s objectives were to use the ROTEM method, which hasn’t been examined in the literature before, to determine whether procoagulant states exist in psoriasis patients.

## Patients and methods

### Study design

We conducted a pilot, prospective and observational study. The study included patients with moderate and severe forms of chronic plaque psoriasis, who were treated at the Department of Dermatology of a tertiary care hospital. The dermatologist examined every patient to determine the Psoriatic Area Severity Index (PASI). We performed the study during the period from 1 January 2022 to 31 December 2023. Patients were enrolled if they met inclusion criteria - age over 18 years, signing an informed consent to participate in the study and moderate and severe plaque psoriasis with PASI > 10 evaluated by the dermatologist. Exclusion criteria were the presence of inherited/acquired bleeding disorder, recent venous or arterial thromboembolic events, uncontrolled arterial hypertension, severe kidney disease (stage 3B– 5 of chronic kidney disease or stage B-C of acute kidney injury), severe liver disease (liver cirrhosis Child B-C or acute liver injury with hepatic encephalopathy and/or severe loss of liver function), pregnancy, patient’s refusal to participate in the study and the use of acetylsalicylic acid, nonsteroidal anti-inflammatory drug or anticoagulant/antiaggregant. None of our patients and healthy control group were using oral contraceptives.

The control group consists of sex-matched healthy blood donors, without known autoimmune and immunologically mediated diseases, examined by the dermatologist to exclude psoriasis, aged 18 years or older, who did not use anticoagulant or antiplatelet therapy, and without severe liver and kidney disease. Patients as well as healthy individuals included in the control group agreed to be included in the study, were instructed about the nature of the study and signed an informed consent to participate in the research and blood sampling.

Along with the clinical data and demographics of the patient group, which comprise gender, age, smoking, previous myocardial infarction, deep vein thrombosis, embolism to arteria pulmonalis, psoriatic arthritis, Body Mass Index (BMI), atrial fibrillation, metabolic dysfunction-associated fatty liver disease, type 2 diabetes mellitus, asthma bronchial, depression, autoimmune thyroiditis, metabolic syndrome, arterial hypertension and dyslipidemia. The patients were examined for a complete blood count, prothrombin time (PT), active partial thromboplastin time (aPTT), fibrinogen, serum creatinine, estimated glomerular filtration rate (eGFR), total bilirubin, total proteins, alanine aminotransferase and ROTEM was also performed to identify any procoagulation condition.

The local ethical committee reviewed and approved the study protocol (Jessenius Faculty of Medicine in Martin, Comenius University in Bratislava, decision protocol No. EK 68/2020).

### Sample collection

Venous blood for examination was taken from the patients during their outpatient visit at 7:00 AM. Blood collection was carried out in a tube with 3.2% sodium citrate by trained staff using the antecubital technique. Blood was stored at room temperature and samples were processed within 1 h of collection. Blood samples of the control group were collected using the same technique, at the same time. Also, these samples were stored at room temperature and examined within 1 h of collection. The examination of the blood sample was carried out at the Department of Hematology and Transfusiology.

### ROTEM analysis and test principles

The samples were examined by rotational thromboelastometry within 1 h of collection by a specialized worker on a ROTEM Gamma (Pentapharm GmbH, Munich, Germany) using INTEM, EXTEM and FIBTEM reagents in a group of psoriatic patients as well as in a group of healthy donors in accordance with the manufacturer’s recommendations.

The basis of the device is a cuvette (cylindrical container) with a volume of 0.34 ml, which does not move [13]. The ROTEM test uses whole blood with the addition of citrate, while calcium is used to activate the sample. It is advisable to examine within 2 h of collection because the stability of the sample is ensured in this time window. During the examination, the cuvette contains a mixture of whole blood (0.3 ml), calcium chloride (0.02 ml) and activator (0.02 ml). The oscillating rotational movement is performed by the mandrel, which is firmly fixed to prevent the transmission of shocks from the surroundings of the device. The mandrel oscillates on the ball mechanism by 4° 75‘ every 6 s with constant force input. If there is no coagulum formation in the mixture, the movement of the mandrel is not blocked. At the moment when fibrin fibers begin to form in the cuvette, the movement of the mandrel is inhibited due to the increase in resistance against the fibrin fibers. These movement changes are transformed into an electrical signal through the optical system and the result is a thromboelastometric curve. If the rotation of the spindle is not blocked, the amplitude is 0 mm (non-coagulated blood). Conversely, the moment without mandrel rotation is converted into a graphical result as an amplitude of 100 mm (maximum possible clot strength). Based on the resulting thromboelastometric curve, we can visually evaluate coagulation from the formation of a clot through its enlargement to lysis in the process of fibrinolysis.

The EXTEM test allows examination of the external clotting pathway. It is essential to add a reagent containing the activator of the external pathway– thromboplastin. The INTEM test allows examination of the internal clotting pathway, a reagent containing the activator of the internal pathway - ellagic acid is used. The FIBTEM assay uses a reagent containing a platelet inhibitor - cytochalasin D. This test allows the examination of functional fibrinogen [20].

We measured clotting time (CT - representing the time data in seconds from the initiation of the test to the detection of the first fibrin fibres with an amplitude of 2 mm), clot formation time (CFT - is the time in seconds from CT, or amplitude of 2 mm to an amplitude of 20 mm), maximum clot firmness (MCF - maximum clot strength in millimeters), amplitude at 10 min post-CT (A10), amplitude at 20 min post-CT (A20), and alpha angle (describes the patient’s coagulability status). Shorter CT, CFT and bigger MCF, A10, A20, and alfa angle were considered a pro-coagulation condition. Each of these parameters was evaluated in INTEM, EXTEM and FIBTEM tests.

### Statistical analysis

The results of the study were processed by statistical software Jamovi v2.3.28.0. and R software, ver. 4.0.5. Continuous variables were summarized by mean and median; variability was summarized by standard deviation, interquartile range. Discrete variables were summarized in numbers and percentages. A triplot consisting of (1) Boxplot, overlaid with swarm plot, (2) density plot, and (3) quantile-quantile plot with bootstrap constructed 95% confidence band was used to check the distribution of continuous variables. The data were tested by the normality test (Shapiro-Wilk test) to evaluate the distribution of the data. In the case of normal data distribution, the statistical significance of the result was assessed by Student’s t-test in the case of abnormal data distribution, the Mann-Whitney *U*-test was used to determine statistical significance. Nominal variables were tested using the chi-square test. A Hampel filter was used to remove outliers. A linear mixed regression model with response with transformed power was used to model the association between response and predictors. We consider a value of *p* < 0.05 to be statistically significant.

## Results

### Patients and controls

We conducted a pilot, prospective, observational study in which we enrolled 62 patients with moderate or severe chronic plaque psoriasis with a PASI > 10. During the duration of the study, 31 men and 31 women were involved with the average age 51 years (±13 years), ranging from 19 years to 85 years. The control group consisted of 61 healthy blood donors (34 men; average age of 42 years (±9 years; ranging from 22 to 64 years). In the group of psoriatic patients, 16 patients (25.8%) were smoking, 1 patient (1.6%) had a history of previous myocardial infarction, 3 patients (4.8%) had a history of previous deep vein thrombosis or pulmonary embolism, on the other hand in the control group 16 patients (25.8%) were smoking. None had a previous myocardial infarction, deep vein thrombosis or pulmonary embolism. Almost 39% of patients (*n* = 24) with psoriasis had also psoriatic arthritis. BMI in psoriatic patients was 29.5 kg/m2 (± 6.1) and in the control group, BMI was 25.5 kg/m2 (± 5.3). Basic demographic information did not show any discernible differences between the group of healthy controls and patients with moderate or severe psoriasis (Table 1).

**Table 1:** Basic demographic and therapy in patients with moderate or severe psoriasis and in healthy donors

	Patients with moderate or severe psoriasis	Healthy donors
Number of patients	62	61
(men / women)	(31 / 31)	(34 / 27)
Age (years)	51 (19–85)	42 (22–64)
PASI	13.2 ± 0.3	N/A
CRP (mg/l)	3.7 ± 0.4	0.5 ± 0.2
IL-6 (ng/l)	3.3 ± 0.3	0.6 ± 0.4
Smoking (%)	16 (25.8%)	16 (25.8%)
Previous myocardial infarction (%)	1 (1.6%)	0 (0%)
Previous deep vein thrombosis / embolism to arteria pulmonalis (%)	3 (4.8%)	0 (0%)
Psoriatic arthritis (%)	24 (38.7%)	0 (0%)
Body Mass Index (kg/m2)	29.5 ± 6.1	25.5 ± 5.3
Atrial fibrillation (%)	3 (4.8%)	0 (0%)
Metabolic dysfunction-associated fatty lived disease (%)	36 (58.1%)	N/A
Type 2 diabetes mellitus (%)	8 (12.9%)	0 (0%)
Asthma bronchial (%)	6 (9.7%)	0 (0%)
Depression (%)	6 (9.7%)	0 (0%)
Autoimmune thyroiditis (%)	4 (6.5%)	0 (0%)
Metabolic syndrome (%)	29 (46.77%)	0 (0%)
Arterial hypertension (%)	35 (56.45%)	0 (0%)
Dyslipidaemia (defined as a value of triacylglycerols above 1.7 mmol/l or HDL-C values below 1 mmol/l in men or below 1.3 mmol/l in women) (%)	48 (77.42%)	0 (0%)
ACE inhibitors, AT1RB (%)	27 (43.54%)	0 (0%)
Lipid-lowering drugs (%)	6 (9.68%)	0 (0%)
Oral antidiabetic drugs (%)	6 (9.68%)	0 (0%)
ARBs (%)	3 (4.84%)	0 (0%)
Calcium channel blockers (%)	14 (22.58%)	0 (0%)
Diuretics (%)	12 (19.35%)	0 (0%)
Betablocker (%)	9 (14.52%)	0 (0%)
Levotyroxin (%)	3 (4.84%)	0 (0%)
Immunosuppressant drugs (%)	47 (75.80%)	N/A
Topical treatment (%)	12 (19.35%)	N/A
Retinoids (%)	2 (3.23%)	N/A
Phototherapy (%)	1 (1.61%)	N/A
Serum creatinine (µmol/L)	68.0 ± 16.1	78.4 ± 15.4
Calculated GFR– Cocroft Gault (ml/min./1.73 m2)	98.2 ± 25.5	81.4 ± 19.8
Total bilirubin (mmol/L)	12.3 ± 4.5	11.2 ± 3.2
Total serum protein (g/l)	58.1 ± 8.2	61.9 ± 6.7
Alanine aminotransferase (ukat/L)	0.51 ± 0.02	0.37 ± 0.03
Haemoglobin (g/L)	145.9 ± 38.2	140.2 ± 29.8
Platelet count (x10^9^/l)	247.2 ± 56.3	254.4 ± 52.2
Fibrinogen (g/l)	3.7 ± 0.7	2.8 ± 0.6
INR	0.95 ± 0.07	0.96 ± 0.06
APTT - ratio	1.04 ± 0.12	1.07 ± 0.08
APTT (s)	30.3 ± 3.4	31.0 ± 2.3
PT (s)	11.2 ± 1.2	11.6 ± 1.2
TT (s)	13.9 ± 1.3	14.3 ± 1.3

PASI – Psoriatic Area Severity Index, CRP – C– reactive protein, IL– interleukin, ACE– angiotensin-converting enzyme, AT1R – AT1 receptor, ARBs – angiotensin receptor blockers, INR– International Normalized Ratio, N/A– not applicable, APTT– Activated Partial Thromboplastin Time, PT– Prothrombin Time, TT– Thrombin Time

### ROTEM analysis

In the performed ROTEM analysis patients with moderate or severe psoriasis were compared with healthy controls (Table 2). When comparing the parameters of the EXTEM test in patients with psoriasis compared to the control group, significant differences were found in CT-EXTEM (74.8 ± 1.4 versus 69.7 ± 1.4 s; *p* < 0.05), MCF-EXTEM, A10-EXTEM, A20-EXTEM and alpha-EXTEM parameters.

**Table 2:** ROTEM analysis in patients with moderate or severe psoriasis vs. healthy donors

ROTEM^®^ (parameter-test)	Patients with moderate or severe psoriasis	Healthy controls	*p* value
CT-EXTEM^®^ (s)	74.8 ± 1.4	69.7 ± 1.4	*p* < 0.05
CFT-EXTEM^®^ (s)	73.6 ± 1.9	75.9 ± 2.0	*p* = 0.426
MCF-EXTEM^®^ (mm)	66.2 ± 0.5	64.3 ± 0.6	*p* < 0.05
A10-EXTEM^®^ (mm)	58.8 ± 0.6	56.7 ± 0.7	*p* < 0.05
A20-EXTEM^®^ (mm)	65.0 ± 0.5	62.8 ± 0.6	*p* < 0.01
α-EXTEM^®^ (°)	75.3 ± 0.3	74.2 ± 0.4	*p* = 0.054
CT-INTEM^®^ (s)	180.0 ± 3.8	191.3 ± 4.8	*p* = 0.071
CFT-INTEM^®^ (s)	73.6 ± 2.1	82.1 ± 2.7	*p* < 0.05
MCF-INTEM^®^ (mm)	62.5 ± 0.6	62.2 ± 0.7	*p* = 0.705
A10-INTEM^®^ (mm)	56.0 ± 0.7	53.5 ± 0.7	*p* < 0.01
A20-INTEM^®^ (mm)	62.1 ± 0.5	60.3 ± 0.6	*p* < 0.05
α-INTEM^®^ (°)	74.4 ± 0.5	71.1 ± 0.6	*p* < 0.001
CT-FIBTEM^®^ (s)	67.3 ± 1.2	62.8 ± 1.2	*p* < 0.05
CFT-FIBTEM^®^ (s)	306.7 ± 14.6	261.0 ± 14.9	*p* < 0.05
MCF-FIBTEM^®^ (mm)	18.1 ± 0.6	16.8 ± 0.6	*p* = 0.103
A10-FIBTEM^®^ (mm)	16.9 ± 0.5	15.6 ± 0.5	*p* = 0.09
A20-FIBTEM^®^ (mm)	17.6 ± 0.6	16.4 ± 0.6	*p* = 0.114
α-FIBTEM^®^ (°)	71.0 ± 0.7	68.7 ± 0.7	*p* < 0.05

CFT– clot formation time, MCF - maximum clot firmness, A– amplitude, CT– clotting time

When INTEM test was used, significant differences were found in CFT-INTEM, A10-INTEM, A20-INTEM and alpha-INTEM parameters (Table 2).

Finally, for FIBTEM testing, significant differences were observed CT-FIBTEM, CFT-FIBTEM (CT-FIBTEM 67.3 ± 1.2 versus 62.8 ± 1.2 s; *p* < 0.05; CFT-FIBTEM 306.7 ± 14.6 versus 261.0 ± 14.9 s; *p* < 0.05), and alpha-FIBTEM.

In correlation analysis (Table 3), in patients with a higher platelet count, A10-INTEM, A20-INTEM, MCF-INTEM, α-INTEM were higher (*r* = 0.728, *p* < 0.001; *r* = 0.683, *p* < 0.001; *r* = 0.643, *p* < 0.001; *r* = 0.674, *p* < 0.001) and CFT-INTEM was shorter (*r*=-0.674, *p* < 0.001). In psoriatic patients with higher leukocytes, A20-INTEM and MCF-INTEM were higher (*r* = 0.326, *p* = 0.01; *r* = 0.335, *p* = 0.008). Using the EXTEM test, higher levels of platelet count were associated with higher A10-EXTEM, A20-EXTEM, MCF-EXTEM, α-EXTEM (*r* = 0.652, *p* < 0.001; *r* = 0.641, *p* < 0.001; *r* = 0.607, *p* < 0.001; *r* = 0.536, *p* < 0.001) and shorter CFT-EXTEM (*r*=-0.584, *p* < 0.001). In psoriatic patients with higher leukocytes, A10-EXTEM, A20-EXTEM and MCF-EXTEM were bigger (*r* = 0.372, *p* = 0.003; *r* = 0.380, *p* = 0.002; *r* = 0.365, *p* = 0.003). In the FIBTEM assay, as platelet count increases, the A10-FIBTEM, A20-FIBTEM and MCF-FIBTEM are getting higher (*r* = 0.476, *p* < 0.001; *r* = 0.457, *p* < 0.001; *r* = 0.419, *p* < 0.001). We detected a similar situation with leukocytes and A10-FIBTEM, A20-FIBTEM, MCF-FIBTEM (*r* = 0.342, *p* = 0.007; *r* = 0.338, *p* = 0.008; *r* = 0.359, *p* = 0.005) and with CRP and A10-FIBTEM, A20-FIBTEM and MCF-FIBTEM (*r* = 0.373, *p* = 0.003; *r* = 0.347, *p* = 0.006; *r* = 0.334, *p* = 0.009).

**Table 3:** Linear regression model and variables with a major effect on ROTEM parameters

ROTEM^®^ (parameter-test)	Variables	*R*	*p*-value
A10-INTEM	PLT	0.728	*p* < 0.001
A20-INTEM	PLT LEU	0.683 0.326	*p* < 0.001 *p* = 0.01
CFT-INTEM	PLT	-0.674	*p* < 0.001
MCF-INTEM	PLT LEU	0.643 0.335	*p* < 0.001 *p* = 0.008
α-INTEM	PLT	0.674	*p* < 0.001
A10-EXTEM	PLT LEU	0.652 0.372	*p* < 0.001 *p* = 0.003
A20-EXTEM	PLT LEU	0.641 0.380	*p* < 0.001 *p* = 0.002
CFT-EXTEM	PLT	-0.584	*p* < 0.001
MCF-EXTEM	PLT LEU	0.607 0.365	*p* < 0.001 *p* = 0.004
α-EXTEM	PLT	0.536	*p* < 0.001
A10-FIBTEM	PLT LEU CRP	0.476 0.342 0.373	*p* < 0.001 *p* = 0.007 *p* = 0.003
A20-FIBTEM	PLT LEU CRP	0.457 0.338 0.347	*p* < 0.001 *p* = 0.008 *p* = 0.006
MCF-FIBTEM	PLT LEU CRP	0.419 0.359 0.334	*p* < 0.001 *p* = 0.005 *p* = 0.009

CRP– C– reactive protein, PLT– platelet count, CFT– clot formation time, MCF - maximum clot firmness, A - amplitude, LEU - leukocytes

Finaly, linear regression models were used to control possible confounding factors. In this analysis, age, smoking status, history of previous thromboembolism, BMI, atrial fibrillation, metabolic dysfunction-associated fatty liver disease, type 2 diabetes, metabolic syndrome, arterial hypertension and dyslipidemia, serum creatinine, eGFR, total bilirubin, total plasma proteins, and alanine aminotransferase levels were considered as variables. This analysis did not reveal any significant impact of upper-mentioned variables on examined ROTEM parameters.

## Discussion

The viscoelastic properties of a clot can be measured in real-time during clot formation, stability, and destruction using a viscoelastic approach ROTEM. In addition to thromboelastography, ROTEM is a novel coagulation test that uses whole blood samples rather than simply blood plasma, in contrast to basic coagulation tests like PT and aPTT, which evaluate just the certain part of the clotting process. The benefit of the ROTEM method is that it allows for direct examination at the patient’s bed; as a result, it is regarded as a bedside or point-of-care method with the availability of rapid findings, enabling a fast and adequate therapeutic intervention. This method has found its application in liver surgery, cardiac surgery, obstetrics, and traumatology; however, the use of ROTEM in dermatology is very limited. Only one study is mapping rotational thromboelastometry profiles in children with chronic spontaneous urticaria. The authors compared 24 children with active chronic spontaneous urticaria and age-matched and sex-matched 30 healthy control participants using INTEM and EXTEM assays. No significant difference between these two groups was found [21]. More studies are present in rheumatology studying the impact of Bechet diseases [22, 23]primary Sjogren syndrome [24] and rheumatoid arthritis [25] on the ROTEM parameters. Our literature search did not find studies dealing with the use of ROTEM in patients with psoriasis, and our study is the first of its kind to evaluate hemostatic disturbances using ROTEM in psoriatic patients. Therefore we are unable to compare our results with other studies.

It is documented by studies and meta-analyses that psoriasis is associated with an increased risk of venous and arterial thromboembolism [6–8]. It is believed that this predisposition is multifactorial and consists of systemic inflammation, vascular endothelial activation and platelet activation. Dysregulation can lead to chronic, systemic inflammation or even thrombotic complications. If inflammation is not controlled, it can turn into chronicity, which, on the other hand, can result in the activation of the coagulation cascade, primarily based on pro-inflammatory cytokines. A study by Visser et al. showed that psoriatic patients displayed a biomarker profile that reflected systemic inflammation, endothelial activation, and increased platelet activity [26]. Our study demonstrates a significant shift toward a hypercoagulable status in patients with psoriasis compared to healthy controls.

As mentioned before, there is no data on the coagulation status assessed with ROTEM in patients with psoriasis. Our pilot study aimed to explore the hemostatic alterations in patients with severe psoriasis using rotational thromboelastometry (ROTEM), a method not previously examined in this context. In our study, we described significantly longer CT-EXTEM^®^, CT-FIBTEMⓇ, CFT-FIBTEMⓇ, shorter CFT-INTEMⓇ and significantly bigger MCF-EXTEMⓇ, A10-EXTEMⓇ, A20-EXTEMⓇ, A10-INTEMⓇ, A20-INTEMⓇ, α-INTEMⓇ and α-FIBTEMⓇ in psoriatic patients. These findings can be interpreted as a shift toward a procoagulant state in patients with severe psoriasis (except of prolonged CT-EXTEM which, in general, indicates hypocoagulation when the coagulation is activated through the extrinsic pathway). As mentioned, there is no other study examining ROTEM pattern in psoriasis, so there is no study for result comparation. Therefore, right know, we are not able to explain this inconsistences in our observation. In our previous study [12]we have described increased MCF (which, in general, represent a more strength clot– hypercoagulation) in patients with acute myocardial infarction on dual antiplatelet therapy (DAPT). In this study, other ROTEM parameters suggested hypocoagulation, as was expected. Although MCF is generally a marker of hypercoagulation, we do not believe that patients on DAPT would have a stronger clot. It is more probable that in this particular scenario - DAPT, the increased MCF would probably indicate more disorganized clot. Concluding, although the majority of observed changes in ROTEM parameters suggest hypercoagulation, these changes might be more complex for understanding, and more studies will be needed to interpret ROTEM pattern in psoriasis.

In a linear regression model we observe the correlation of ROTEM parameters with platelets, leukocytes and CRP. As is generally known, psoriasis is a chronic immune-mediated inflammatory disease in which IL-23 and pathological Th17 cell differentiation play crucial roles [1, 2]. It has been shown that Platelet-activating factor induces Th17 cell differentiation [26] and that peripheral blood mononuclear and polynuclear cells (leukocytes) express IL-23 in an in-vitro model [27]. The connection between increased (sub-clinical) inflammation and thrombosis is generally known and accepted [28]and was also described in the settings of psoriasis [29]. In addition, CRP is a useful marker of psoriasis activity [2]. Although several studies reported the association between platelets and platelet markers with psoriasis activity and risk of thrombotic events in patients with psoriasis [10]; right know it is unclear, whether leucocytes, platelets and CRP could be used as a marker of hypercoagulation in psoriatic patients and further research regarding this issue is still needed.

It is important to add more data analyzing ROTEM parameters in psoriatic patients because changes in the CT and CFT-FIBTEM^®^ shift the coagulation status to hypocoagulation. We can hypothesize that it can be associated with the change in fibrin microstructure [30]. The procoagulant tendency observed in our study aligns with the growing body of evidence that psoriasis is associated with an increased risk of thrombotic events. Chronic systemic inflammation, endothelial dysfunction, and platelet activation likely contribute to these haemostatic changes. Previous research has demonstrated increased platelet activity and endothelial activation in psoriatic patients, further supporting our findings [31, 32]. While the ROTEM method provides valuable real-time insights into the coagulation process, its application in dermatology, particularly in psoriasis, is limited. Our study is the first to document significant hemostatic disturbances in psoriatic patients using ROTEM, which emphasizes the potential of this method to enhance the understanding of thrombotic risks in psoriasis. These findings suggest that hemostatic alterations in psoriasis are multifactorial and require further investigation to understand the underlying mechanisms better.

### Limitations

Despite the novelty of this study, several limitations should be mentioned. First of all, our pilot study was observational and had a relatively small sample size, which may affect the results. The larger study studies with a more diverse population are needed to confirm our findings. Furthermore, the individuals in our study had only moderate to severe psoriasis; hence, we were unable to investigate potential variations in hemostatic profiles in less severe cases. In addition, there were differences in age and BMI between patients and controls, and this differences could possibly impact the observation of hypercoagulation. It was previously demonstrated that obesity (with or without type 2 diabetes) [33–35] and an increase in age can be also connected with shift towards hypercoagulable ROTEM profile [36]. However, considering the fact that the prevalence of obesity, type 2 diabetes or prediabetes and metabolic syndrome is high in psoriasis (sometimes metabolic syndrome is considered to be a psoriasis-associated disease) [2]it would be difficult to find sample of patients with moderate to severe psoriasis who will have a BMI match with healthy controls. The same difficulties would be probably present with age-matching, as a mean age of onset of psoriasis is 33 years [2]. Finally, we were unable to ascertain the clinical significance of these hemostatic alterations in relation to actual thrombotic events due to the lack of long-term follow-up data.

## Conclusion

Our study is the first to prove statistically significant changes in hemostasis in patients with severe psoriasis assessed with ROTEM, revealing a shift toward a procoagulant state, compared to healthy blood donors. It is the very first study to show disturbances in ROTEM parameters in test INTEM, EXTEM and FIBTEM. These findings contribute to the understanding of thrombotic risks in psoriasis and suggest that ROTEM could serve as a useful tool for assessing coagulation disturbances in this population. Further studies are needed to confirm our results and explore the clinical relevance of these changes.

## Data Availability

All the data are available at the Corresponding Author upon a reasonable request.
